# The Burden of Worry: Fear of Cancer Recurrence Across Bladder Cancer Survivorship Phases—A Cross-Sectional Analysis

**DOI:** 10.3390/jcm15083116

**Published:** 2026-04-19

**Authors:** Dor Golomb, Sébastien Simard, Alon Eisner, Yuval Avda, Fahed Atamna, Orit Raz

**Affiliations:** 1 Department of Urology, Assuta Ashdod University Hospital, 7 Harefua St., Ashdod 7747629, Israel; 2 Department of Health Sciences, Université du Québec à Chicoutimi, Saguenay, QC G7H 2B1, Canada; sebastien.simard@criucpq.ulaval.ca

**Keywords:** fear of cancer recurrence (FCR), bladder cancer, non-muscle invasive bladder cancer (NMIBC), survivorship

## Abstract

**Objective:** To characterize the distribution of FCR severity across survivorship time intervals in bladder cancer survivors. **Methods:** A cross-sectional study of 79 patients utilized the validated 9-item FCR Inventory-Short Form (FCRI-SF) to assess overall FCR severity. Primary analysis employed Spearman’s correlation coefficient to evaluate the relationship between time elapsed since the first procedure and total FCR scores. Patients were stratified into four temporal groups (<1, 1–2, 2–5, and >5 years). Inter-group variability in FCR scores was assessed using Levene’s test for equality of variances. Subgroup analyses compared FCR scores across clinical subgroups, including tumor grade and smoking history, using the Mann–Whitney U test. Multivariate logistic regression identified independent predictors of clinically significant FCR (total score ≥13). **Results:** Median patient age was 72.0 years (IQR 66.0–78.0), with a median of 24.0 months post-diagnosis. Clinically significant FCR (score ≥13) was prevalent in 55.7% of the cohort. Spearman correlation analysis revealed no significant relationship between months elapsed and FCR severity (rho = 0.068, *p* = 0.552). Patients in the 12–24 month window exhibited the highest variability (Levene’s test, *p* = 0.058), representing a period of clinical divergence. High-Grade disease and smoking cessation motivated by diagnosis were associated with higher FCR scores. In the multivariate logistic regression model, history of tumor recurrence was the sole independent predictor of clinically significant FCR (aOR 3.28, 95% CI 1.11–9.68, *p* = 0.031), whereas age and gender were not significantly associated. **Conclusions:** FCR severity did not demonstrate a significant association with time elapsed since diagnosis in this cross-sectional sample. The 1–2 year interval demonstrated greater inter-individual variability in FCR scores. Findings highlight the need for long-term, structured survivorship support, particularly targeting the 12–24 month post-diagnosis window.

## 1. Introduction

Bladder cancer is the tenth most common cancer worldwide, characterized by a clinical course that often requires decades of intensive medical surveillance due to high recurrence and progression rates [1]. For patients with Non-Muscle Invasive Bladder Cancer (NMIBC), the standard of care involves intravesical instillations, regular cystoscopies and repeated surgical interventions [2]. While these protocols are effective for oncological control, they impose a significant and sustained psychological burden on the survivors [3].

Among the various psychological challenges faced by cancer survivors, FCR is frequently cited as the most prevalent and distressing concern [4]. FCR is defined as the fear, dread, or worry that cancer will return or progress in the same organ or another part of the body [5]. Traditionally, psychological distress in oncology is viewed through an “adaptation model,” where anxiety is highest at diagnosis and gradually dissipates as the patient remains cancer-free [6]. However, the unique clinical trajectory of bladder cancer may prevent such habituation. In NMIBC, the objective risk of recurrence does not necessarily decline; rather, the cumulative probability of new events and the need for more aggressive interventions may increase over time [7]. This chronic threat is reinforced by the “re-traumatization” of invasive follow-up procedures and the persistent medical reality that recurrence is an expected clinical event rather than a rare failure. Consequently, FCR in this population may not follow a linear decay. For patients with high baseline anxiety, the passage of time may fail to provide relief because the “time-at-risk” continues to expand, often alongside an increase in the number of prior recurrences or the severity of subsequent treatments. Despite this, there is a paucity of data regarding the temporal trajectory of this fear in urological oncology.

The objective of this study was to evaluate the relationship between the time elapsed since the initial surgical procedure and the overall severity of FCR. By aligning psychological distress with medical data—such as disease severity and the persistent nature of urological surveillance—we aim to explore whether the “chronic” nature of bladder cancer creates a unique plateau or resurgence in fear, thereby identifying the interval of heightened inter-individual variability for psychological intervention in the survivorship continuum. This hypothesis informed our exploratory study design but cannot be confirmed from cross-sectional observations alone.

## 2. Patients and Methods

### 2.1. Study Design and Population

This cross-sectional study was conducted as part of a larger clinical initiative to evaluate the long-term psychological impact of urological cancer surveillance. Participants were recruited consecutively by the urology clinical team during their routine follow-up visits at a single tertiary medical center. To be eligible, patients were required to have a confirmed histological diagnosis of a bladder tumor and a history of at least one surgical procedure. Upon recruitment, patients completed the FCRI questionnaire in the outpatient clinic. The questionnaire was administered primarily via a paper-based version. A research assistant was present during the completion of the FCRI to provide technical assistance and ensure that the unidimensional severity measure was fully understood and completed by the participants. A formal a priori power calculation was not performed; this study was conducted as part of a clinical audit initiative.

### 2.2. Data Collection

Clinical and demographic data were retrospectively retrieved from electronic medical records and supplemented by patient interviews. Demographic variables included age, gender, marital status, body mass index (BMI), education level, and smoking history. Clinical variables included baseline tumor staging at diagnosis and the administration of adjuvant therapy. Beyond the initial diagnosis, we assessed the temporal trajectory using ‘Time Since First Procedure,’ defined as the interval between the primary surgical intervention and psychological assessment. Tumor grade was categorized as Low-Grade (LG) or High-Grade (HG). Smoking history was specifically examined for patients who successfully ceased smoking due to their tumor diagnosis, as this represents a behavioral indicator of disease awareness and psychological engagement with the illness.

### 2.3. Psychological Assessment

FCR was assessed using the FCRI-SF, a unidimensional scale designed to quantify the overall severity of psychological distress. Patients responded to nine specific items that capture various manifestations of FCR severity, including the intensity of worry (e.g., “I am afraid of cancer recurrence”), the frequency of intrusive cognitions (e.g., “How often do you think about the possibility of cancer recurrence?”), and the perceived risk of future oncological events. Each manifestation was scored on a Likert-type scale ranging from 0 to 4. In alignment with the psychometric properties of the short version, these items were analyzed as indicators of a single underlying dimension of severity rather than independent subscales. This approach ensures that the multifaceted nature of the fear—encompassing emotional, cognitive, and risk-based elements—is captured within a total severity index that reflects the comprehensive burden on the survivor. Each of the nine items is scored on a Likert scale from 0 to 4, yielding a total continuous score ranging from 0 to 36. In accordance with established clinical validation for this instrument, a total score of ≥13 was utilized as the cutoff threshold to indicate clinically significant FCR for our multivariate models.

### 2.4. Statistical Analysis

Descriptive statistics were used to summarize patient demographics and clinical characteristics. The primary analysis evaluated the relationship between time elapsed since the initial procedure (measured in months) and FCR severity (total score ranging from 0 to 36) as continuous variables using Spearman’s rank correlation coefficient, given the non-normal distribution of both variables. The FCRI-SF was analyzed as a unidimensional total severity score in accordance with the original psychometric validation, which characterizes the short form as yielding a single severity index rather than subscale scores. Subgroup analyses were performed to identify clinical and behavioral predictors of FCR. Median FCR scores were compared across clinical subgroups—including tumor grade (LG vs. HG), adjuvant therapy status, and smoking history using the Mann–Whitney U test for all pairwise comparisons. Effect sizes for all Mann–Whitney comparisons are reported as rank-biserial correlation coefficient (r). Variance in FCR scores across temporal groups was assessed using Levene’s test for equality of variances. Multivariate logistic regression was employed to identify independent predictors of clinically significant FCR (total score ≥13). Covariate selection was guided by clinical relevance and a target events-per-variable ratio of ≥10. Number of prior surgical procedures was included as an additional covariate. The final model included the following five covariates: age (continuous), gender (male vs. female), history of tumor recurrence (yes vs. no), tumor grade (Low-Grade vs. High-Grade), and number of prior surgical procedures (yes vs. no). The achieved events-per-variable ratio was 8.4, which approximates the recommended threshold and should be interpreted accordingly. Although age, gender, tumor grade, and recurrence history were conceptually identified as clinically relevant a priori, the final covariate set was not formally pre-specified prior to data collection; it was confirmed following data review. This selection should therefore be understood as post hoc and data-informed rather than prospectively pre-registered, and results should accordingly be treated as hypothesis-generating. Results are reported as adjusted odds ratios (aOR) with 95% confidence intervals (95% CI). A two-sided *p*-value of <0.05 was considered statistically significant. Model fit was assessed using the Nagelkerke R^2^, which quantifies the proportion of variance in the outcome explained by the model, and the Hosmer–Lemeshow goodness-of-fit test, which evaluates the agreement between observed and predicted outcomes across risk strata; a non-significant result (*p* > 0.05) indicates adequate model calibration. The revised model yielded a Nagelkerke R^2^ of 0.131, indicating that the included covariates explain approximately 13% of the variance in clinically significant FCR. The Hosmer–Lemeshow test demonstrated adequate model calibration (χ^2^(6) = 5.929, *p* = 0.431), with no significant discrepancy between observed and predicted outcome frequencies across risk groups.

## 3. Results

**Figure 1:**
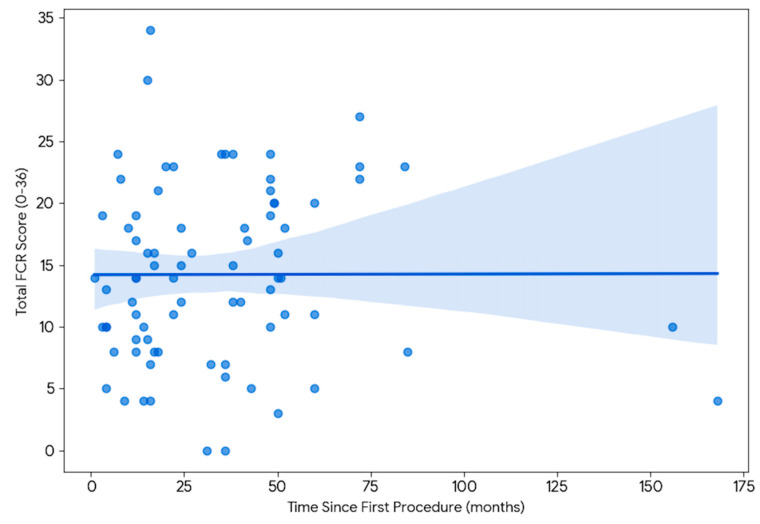
Spearman correlation between time since first procedure (months) and total Fear of Cancer Recurrence (FCR) score.

**Figure 2:**
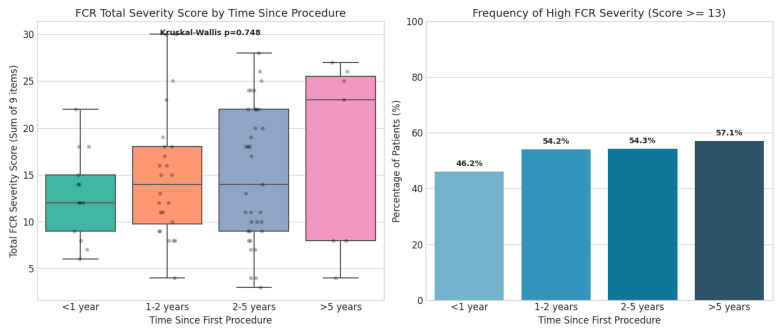
Distribution of Total FCR Severity Scores Across Bladder Cancer Survivorship Phases. Data represent cross-sectional group comparisons at distinct time intervals; this figure should not be interpreted as depicting within-patient longitudinal change.

The analysis included a cohort of 79 patients with a history of bladder tumors. Patient demographics are provided in Table 1. The median age of the participants was 72.0 years (IQR 66.0–78.0). The majority of the study population was male (N = 71, 89.9%). Clinical staging was predominantly NMIBC, with TaLG (N = 56, 70.9%) and T1HG (N = 15, 19.0%) being the most frequent diagnoses observed. The time elapsed since the first surgical procedure ranged from 1 to 168 months, with a median of 24.0 months (IQR 13.0–48.0) for the entire cohort. Using the full 9-item FCRI-SF (total score range 0–36), clinically significant FCR (defined as a total score ≥13) was prevalent in 55.7% of the cohort. While many patients reported manageable levels of worry, this high prevalence indicates that for the majority of survivors, the psychological burden of recurrence remains a dominant and persistent concern long after the initial diagnosis. Correlation analysis using Spearman’s rho revealed no significant association between the length of time since the first procedure and the intensity of FCR (Spearman’s rho = 0.068, *p* = 0.552). No statistically significant association was identified between time elapsed and FCR severity; this finding should be interpreted in the context of limited statistical power to detect small effect sizes. To evaluate the distribution of FCR severity across survivorship phases, patients were categorized into four temporal groups: the early phase of <12 months (N = 20), the intermediate phase of 12–24 month (N = 20), the long-term phase of 24–60 months (N = 32), and the extended survivorship phase of over 60 months (N = 7) (Table 2). Spearman correlation analysis revealed no statistically significant monotonic relationship between months elapsed since the initial procedure and total FCR severity (rho = 0.068, *p* = 0.552) (Figure 1). As this is a cross-sectional study, the following group-level observations reflect differences between independent patient cohorts at distinct time intervals and should not be interpreted as individual-level longitudinal change. While median intensity scores appeared relatively stable across groups, greater inter-individual variability in FCR scores was observed (Figure 2). Patients in the 12–24 month intermediate window exhibited the highest variability in their responses (Levene’s test for equality of variances, *p* = 0.058). Variance differences detected by Levene’s test reflect differences in the spread of scores between groups and should not be interpreted as reflecting distinct psychological states without confirmatory longitudinal data. Furthermore, patients in the extended survivorship group (>5 years) showed higher median frequency-of-thoughts scores compared to the 2–5 year group; this observation is exploratory given the small group size (N = 7). This suggests that long-term survivors may experience a degree of stability in which the cognitive presence of the illness persists even as the acute treatment phase recedes. Subgroup analyses identified specific clinical drivers of FCR severity. Patients with HG disease reported higher median FCR severity scores compared to those with LG disease (Median HG: 16.0, IQR 10.5–19.0; Median LG: 12.5, IQR 9.0–22.0; *p* = 0.5814; rank-biserial r = −0.080). This difference did not reach statistical significance and should be considered hypothesis-generating. Additionally, patients who successfully ceased smoking specifically because of their tumor diagnosis maintained higher persistent FCR scores (Median: 17.0, IQR 12.0–21.5) compared to those who quit prior to diagnosis or never quit (Median: 13.0, IQR 9.0–19.0; *p* = 0.3680; rank-biserial r = −0.155). This difference did not reach statistical significance and should be considered hypothesis-generating. Mann–Whitney U test was used for all statistical comparisons, with rank-biserial r reported as the effect size measure. In the multivariate logistic regression model adjusting for demographic and clinical variables (Table 3), history of tumor recurrence remained the only independent predictor of clinically significant FCR (aOR 3.28, 95% CI 1.11–9.68, *p* = 0.031). The regression model yielded a Nagelkerke R^2^ of 0.131, indicating that the included covariates explain approximately 13% of the variance in clinically significant FCR. The Hosmer–Lemeshow test confirmed adequate model calibration (χ^2^(6) = 5.929, *p* = 0.431). Age was not significantly associated with FCR severity (aOR 1.00 per year, 95% CI 0.97–1.02, *p* = 0.834), and male gender was also not significantly associated (aOR 0.37 for males vs. females, 95% CI 0.07–2.04, *p* = 0.255). These findings suggest that the clinical course of the disease, specifically the event of recurrence, is a more potent driver of psychological distress than baseline demographic factors or other clinical variables examined. Given the borderline events-per-variable ratio (EPV = 8.4) and modest sample size (79 patients, 44 events), these regression results must be interpreted as exploratory and hypothesis-generating rather than confirmatory. The confidence intervals are wide, reflecting considerable uncertainty around the point estimates, and independent replication in adequately powered prospective cohorts is required before definitive conclusions can be drawn. The regression findings reported here are preliminary and should not be used as the basis for definitive clinical conclusions regarding independent predictors of FCR. The wide confidence intervals (e.g., 95% CI 1.11–9.68 for the primary predictor) underscore the uncertainty inherent to this small-sample analysis.

## 4. Discussion

The primary objective of this study was to evaluate the relationship between time elapsed since surgical intervention and the severity of FCR in bladder cancer survivors. While a longitudinal study would be required to definitively map an individual patient’s trajectory, our cross-sectional data do not demonstrate a significant decline in FCR severity with increasing time since diagnosis; however, whether FCR follows a non-linear or plateau trajectory can only be established through a longitudinal study. This model typically suggests that psychological distress peaks at diagnosis and gradually remits as a patient remains cancer-free [8]. In sharp contrast, our findings are consistent with the possibility that FCR does not naturally decline, though the cross-sectional design and limited power preclude a definitive conclusion. With clinically significant FCR (≥13) present in 55.7% of our cohort, our data are consistent with the possibility that the burden of worry does not naturally decay over time for many patients. This interpretation should be considered exploratory, given the cross-sectional design and underpowered sample. The deviation from standard adaptation likely stems from the unique clinical nature of NMIBC. Unlike many solid tumors, where the 5-year mark is colloquially associated with a “cure,” high recurrence rates necessitate lifelong surveillance, preventing patients from reaching psychological closure. Consequently, the clinical course of the disease itself outpaces survivorship duration as the primary driver of distress. In our multivariate analysis, experiencing a tumor recurrence was the sole independent predictor of clinically significant FCR. This suggests that the realization of the “worst-case scenario” validates the patient’s fears, consistent with the possibility that the realization of the ‘worst-case scenario’ may reinforce patients’ fears, though the cross-sectional design does not permit causal inference regarding this mechanism. It should be noted that time since first procedure and time since last recurrence are conceptually distinct exposures. The former captures the full survivorship arc from diagnosis; the latter reflects disease activity recency. Future studies should consider both anchors to disentangle these constructs.

Interestingly, our analysis yielded divergent findings regarding demographic predictors of FCR. While younger age is a well-established predictor of elevated FCR across the broader oncology literature [9], our multivariate model demonstrated no significant association between age and FCR severity. Similarly, gender did not reach statistical significance. This discrepancy may be attributed to the specific demographic profile of bladder cancer patients, who are generally older compared to breast or testicular cancer survivors often cited in FCR research. In this specific population, it appears that the tangible threat of disease recurrence outweighs demographic vulnerabilities. For the clinician, this implies that older age should not be viewed as a protective factor against anxiety; rather, psychological screening is warranted for all patients with a history of recurrence, irrespective of age or gender. Ultimately, these findings suggest that the “watchful waiting” inherent to bladder cancer surveillance creates a state of chronic vigilance. Since our data do not support the assumption that time alone mitigates this distress, urologic oncology teams should consider integrating regular psychological screening into follow-up protocols, recognizing that a patient 10 years post-resection may be just as psychologically vulnerable as one diagnosed yesterday.

Furthermore, Patients in the extended survivorship group showed higher median frequency-of-thoughts scores compared to the 2–5 year group. This group-level observation, albeit exploratory given the small sample size (N = 7), may reflect a ‘cumulative risk’ perception among longer-term survivors, a psychological state often described as “waiting for the other shoe to drop”.

The clinical implications of these findings are significant. Because our data do not demonstrate a natural decline in FCR severity with time, these findings suggest it may not be appropriate to treat FCR as a transient post-diagnosis reaction in this population. Instead, urologists and oncology nurses should incorporate FCR assessments into long-term follow-up protocols. The stability of these scores over a decade indicates that screening remains relevant throughout the entire survivorship continuum, ensuring that patients with persistent high-severity FCR are identified and offered targeted psychological support. The lifelong requirement for intensive medical surveillance in bladder cancer, characterized by regular cystoscopies and repeated surgical interventions, creates a unique clinical environment that may hinder psychological recovery. Unlike other malignancies, where treatment completion leads to a period of non-invasive “watchful waiting,” the standard of care for NMIBC involves frequent, invasive procedures that serve as periodic triggers for FCR. While these follow-up encounters offer temporary reassurance, they often function as a compensatory coping mechanism rather than a path to psychological adaptation. This reliance on medical validation creates a “vicious cycle”: the patient’s fear is momentarily reduced by a clear examination, but they do not learn to manage the uncertainty of their condition independently. Consequently, the need for the medical team’s appraisal to “continue living their life” maintains the future-oriented worry regarding the possibility of cancer returning or its serious consequences. By constantly re-introducing the surgical and diagnostic setting, the surveillance protocol may inadvertently reinforce the chronic nature of this distress, establishing FCR as a persistent clinical entity rather than a transient post-diagnosis reaction. To note, these mechanistic interpretations are speculative in the absence of longitudinal data and are offered as hypotheses for future investigation.

Our findings suggest a necessary shift in the current urological follow-up paradigm, which has traditionally prioritized the physical detection of recurrence through oncological control and surveillance. While medical protocols focus on long-term stability, clinicians must recognize that the first two years post-diagnosis represent a period of heightened psychological vulnerability. During this window, our data revealed a broader peak in the distribution of worry intensity (IQR 1.0–3.2) compared to the early phase. This surge in distress may be linked to a tapering of medical supervision; as the frequency of surveillance cystoscopies begins to decrease, patients may feel less “monitored,” leading to an increase in intrusive thoughts and anxiety regarding their health status. Consistent with the pattern described above, this intermediate window represents a time interval of potential clinical relevance for psychological screening. Integrating standardized assessments into routine follow-up during this transition is essential to ensure that survivors receive the necessary support as they move from acute treatment to long-term surveillance.

## 5. Study Implications

The findings of this study carry direct implications for the structuring of urological follow-up care. Given that FCR does not naturally remit with time, and that tumor recurrence is its sole independent predictor, a reactive model of psychological support—one triggered only by patient complaint or acute distress—is likely to miss the majority of affected survivors. Instead, our data supports the case for embedding a brief, validated screening tool such as the FCRI-SF into routine cystoscopy follow-up visits, particularly during the 12–24 month post-diagnosis window identified here as an interval of greater inter-individual variability. This would require minimal additional clinical burden, as the 9-item instrument can be completed in under two minutes. Positive screens should trigger referral pathways to psycho-oncology services or, where these are unavailable, to structured nurse-led survivorship programs. To note, variance differences detected by Levene’s test should not be interpreted as reflecting distinct psychological states without confirmatory longitudinal data. Urologists and oncology nurses are uniquely positioned to normalize these conversations, framing psychological screening not as an indication of weakness but as a standard component of comprehensive cancer care—analogous to monitoring for biochemical recurrence. Health systems serving bladder cancer populations should consider formalizing this approach within survivorship care plan frameworks, recognizing that the psychological cost of untreated FCR extends beyond individual suffering to encompass reduced treatment adherence, increased healthcare utilization, and diminished quality of life over what may be a decades-long survivorship continuum.

## 6. Study Limitations

Several limitations of this study must be acknowledged explicitly. First, and most fundamentally, the cross-sectional design prevents the establishment of within-patient temporal inference. All comparisons presented reflect group-level observations at distinct time intervals and should not be interpreted as individual-level longitudinal change. While different patient cohorts were compared at various points in their survivorship, individual intra-patient trajectories were not tracked. A prospective longitudinal design would be required to capture the nuanced fluctuations in FCR that may occur in response to specific clinical triggers, such as upcoming surveillance cystoscopies or the diagnosis of a new recurrence. Second, a formal a priori power calculation was not performed, as this study was conducted as part of a clinical audit initiative. Post-hoc analysis indicates that the sample was underpowered to reliably detect small effect sizes. Consequently, the primary null finding—no significant association between time elapsed and FCR severity—should be interpreted with caution and does not constitute evidence of a true absence of association. Time since first procedure may be less closely aligned with current psychological risk than time since last recurrence or recurrence frequency, which may more directly capture the patient’s evolving threat appraisal. This limitation also applies to the subgroup analyses: the observed differences in FCR scores between High-Grade and Low-Grade disease groups, and between patients who ceased smoking due to diagnosis and those who did not, did not reach statistical significance (*p* = 0.58 and *p* = 0.37, respectively) and should be considered hypothesis-generating only, pending confirmation in adequately powered studies. Third, the extended survivorship group (>5 years post-diagnosis) comprised only seven participants. Any observations regarding FCR patterns in this subgroup are exploratory and should not be generalized. The statistical power to detect meaningful differences at this time interval is substantially limited, and this group’s small size precludes firm conclusions regarding late-phase FCR behavior. Fourth, the study population was recruited from a single tertiary academic center and was predominantly male (89.9%). These characteristics limit the external validity of the findings. The results may not generalize to female bladder cancer patients, who represent a clinically and psychologically distinct population, nor to patients managed in community or non-academic settings where surveillance protocols and patient support structures may differ substantially. Fifth, state-dependent assessment bias cannot be excluded. FCR scores were obtained at a single time point during routine outpatient follow-up, and a patient’s score may have been transiently elevated if the assessment coincided with an upcoming surveillance cystoscopy or a period of diagnostic uncertainty. Future studies should document the timing of assessment relative to scheduled procedures to allow for adjustment of this potential confounder. Sixth, treatment burden could not be fully characterized. The specific type of adjuvant intravesical therapy administered, BCG versus chemotherapy, was not uniformly documented in the medical records and could therefore not be included as a covariate. The study sample included patients with NMIBC (TaLG and T1HG) as well as T2HG and other diagnoses. Differences in surveillance burden, BCG exposure, number of prior recurrences, and time since last recurrence across these subgroups may independently influence FCR and were not fully characterized in the present analysis. This heterogeneity limits the precision of conclusions and should be addressed in future studies with more granular clinical phenotyping. Given that treatment modality and intensity may independently influence psychological distress, this represents a notable gap in the current analysis. Seventh, the multivariable logistic regression model was constructed in a sample of 79 patients with 44 events, limiting statistical power and the reliability of adjusted estimates. Results should be considered preliminary and hypothesis-generating; prospective validation in adequately powered cohorts is required before clinical conclusions can be drawn regarding independent predictors of clinically significant FCR. Finally, the number of prior recurrences was retrospectively extracted from electronic medical records and may underrepresent the true recurrence burden for some patients, particularly those with events documented outside the study institution. Although this variable was incorporated into the revised multivariate model, its accuracy as a covariate is contingent on the completeness of the clinical record. Future prospective, multi-center studies with standardized data capture are warranted to validate these findings and more precisely delineate the clinical and psychological predictors of persistent FCR in this population.

## 7. Conclusions

In this cross-sectional study of 79 bladder cancer survivors, FCR severity was not significantly associated with time since the initial procedure. Clinically significant FCR was present in 55.7% of the cohort, suggesting that psychological burden remains prevalent across survivorship phases. History of tumor recurrence was the only independent predictor of clinically significant FCR in the multivariate model. Greater inter-individual variability in FCR scores was observed at the 1–2 year interval, a finding that warrants prospective investigation. These findings support the integration of standardized FCR screening into routine urological follow-up, with particular attention to patients with a history of recurrence.

## Figures and Tables

**Table 1:** Demographic and Clinical Characteristics of the Study Population.

Characteristic	Total Cohort (N = 79)
Age (years), Median [IQR]	72.0 [65.0–77.0]
Gender (Male), n (%)	71 (89.9%)
BMI (kg/m^2^), Median [IQR]	28.0 [25.1–30.4]
Marital Status, n (%)	
Married/Partnered	53 (67.1%)
Single/Other	26 (32.9%)
Smoking History, n (%)	
Ever Smoker	65 (82.3%)
Never Smoker	14 (17.7%)
Smoking cessation due to diagnosis	14 (17.7%)
Baseline Tumor Staging, n (%)	
TaLG	56 (70.9%)
T1HG	15 (19.0%)
T2HG/Other	8 (10.1%)
Current Disease Status, n (%)	
Recurrent Disease	23 (29.1%)
First Manifestation	56 (70.9%)
Time Since First Procedure (months), Median [IQR]	24.0 [13.0–48.0]
Psychiatric History, n (%)	
Anxiety or Depression	10 (12.7%)
Clinically Significant FCR (Total Score ≥ 13), n (%)	44 (55.7%)

BMI—Body Mass Index; kg—Kilograms; IQR—Interquartile Range; FCR—Fear of Cancer Recurrence.

**Table 2:** Fear of Cancer Recurrence (FCR) Scores and Subdomain Measures Across Survivorship Time Intervals.

Measure	Early Phase (<1 yr) N = 20	Intermediate (1–2 yrs) N = 20	Long-Term (2–5 yrs) N = 32	Extended (>5 yrs) N = 7
Total FCR Score (range 0–36)				
Median [IQR]	13.0 [9.8–16.5]	12.5 [8.8–18.2]	15.5 [9.0–22.0]	23.0 [8.0–25.5]
Clinically significant FCR ^a^ (≥13), n (%)	10 (50.0%)	10 (50.0%)	18 (56.2%)	4 (57.1%)
Worry Intensity (items 1–3; range 0–12) ^b^				
Median [IQR]	6.0 [5.0–8.2]	6.5 [4.0–9.5]	8.0 [4.0–10.0]	8.0 [4.5–9.5]
Frequency of Intrusive Thoughts (items 7–9; range 0–12) ^c^				
Median [IQR]	0.5 [0.0–2.0]	1.0 [0.0–4.0]	1.5 [0.0–3.2]	6.0 [0.5–8.0]
Perceived Recurrence Risk (items 4–6; range 0–12) ^d^				
Median [IQR]	6.0 [5.0–7.0]	5.5 [4.0–7.0]	6.0 [4.0–7.0]	7.0 [3.5–7.5]

IQR = Interquartile Range; FCR = Fear of Cancer Recurrence. ^a^ Clinically significant FCR defined as total FCRI-SF score ≥ 13 per established validation threshold. ^b^ Worry Intensity cluster: items “I’m worried or anxious about the possibility of cancer recurrence,” “I am afraid of cancer recurrence,” “I believe it is normal to be worried.” Presented as exploratory descriptive data; the FCRI-SF is validated as a unidimensional instrument and subdomain scores are not validated for independent inference. ^c^ Frequency of Intrusive Thoughts cluster: items “How often do you think about the possibility of cancer recurrence?” “How much time per day do you spend thinking about the possibility of cancer recurrence?” “How long have you been thinking about the possibility of cancer recurrence?” ^d^ Perceived Recurrence Risk cluster: items “When I think about the possibility of recurrence, this triggers other unpleasant thoughts or images,” “I believe I am cured and that the cancer will not come back,” “In your opinion, are you at risk of having recurrence?” Kruskal–Wallis test across all four groups for total FCR score: H = 0.942, *p* = 0.815. Levene’s test for equality of variances: F = 2.605, *p* = 0.058. Group-level comparisons are cross-sectional and should not be interpreted as individual-level longitudinal change. Subdomain clusters (Worry Intensity, Frequency of Intrusive Thoughts, Perceived Recurrence Risk) are presented as descriptive item groupings only and do not represent independently validated subscales. The FCRI-SF is validated as a unidimensional instrument; the total severity score is the primary outcome measure.

**Table 3:** Multivariate Logistic Regression Analysis of Predictors for Clinically Significant Fear of Cancer Recurrence.

Variable	Adjusted OR	95% CI	*p*-Value
History of Tumor Recurrence (Yes vs. No)	3.28	1.11–9.68	0.031
Age (per year increase)	1.00	0.97–1.02	0.834
Male Gender (vs. Female)	0.37	0.07–2.04	0.255
Tumor Grade (High-Grade vs. Low-Grade)	1.56	0.56–4.36	0.400
Number of Prior Surgical Procedures (Yes vs. No)	1.18	0.40–3.54	0.763

## Data Availability

The data presented in this study are available on request from the corresponding author.

## Abbreviations

aOR	Adjusted Odds Ratio
BCG	Bacillus Calmette-Guérin
BMI	Body Mass Index
CI	Confidence Interval
FCR	Fear of Cancer Recurrence
FCRI-SF	Fear of Cancer Recurrence Inventory-Short Form
HG	High-Grade
IQR	Interquartile Range
LG	Low-Grade
NMIBC	Non-Muscle Invasive Bladder Cancer
OR	Odds Ratio
*p*	*p*-value (statistical significance)
rho	Spearman’s Rank Correlation Coefficient
T1HG	Tumor Stage 1, High-Grade
TaLG	Tumor Stage Ta, Low-Grade
T2HG	Tumor Stage 2, High-Grade
